# SARS-CoV-2 infection as a model to study the effect of cinnamaldehyde as adjuvant therapy for viral pneumonia

**DOI:** 10.1186/s12950-023-00364-9

**Published:** 2023-11-20

**Authors:** Bianca Vezzani, Mariasole Perrone, Marianna Carinci, Laura Palumbo, Alberto Tombolato, Denis Tombolato, Claudio Daminato, Valentina Gentili, Roberta Rizzo, Gianluca Campo, Luca Morandi, Alberto Papi, Savino Spadaro, Paolo Casolari, Marco Contoli, Paolo Pinton, Carlotta Giorgi

**Affiliations:** 1 Department of Medical Sciences, Section of Experimental Medicine, University of Ferrara, 44121 Ferrara, Italy; 2 Laboratory of Technologies for Advanced Therapy (LTTA), Technopole of Ferrara, 44121 Ferrara, Italy; 3 CinnaPharm, Castello Di Godego, 31030 Treviso, Italy; 4 Department of Chemical, Pharmaceutical and Agricultural Sciences, University of Ferrara, 44121 Ferrara, Italy; 5 Cardiology Unit, Azienda Ospedaliero-Universitaria Di Ferrara, Ferrara, Italy; 6 Department of Translational Medicine, University of Ferrara, 44121 Ferrara, Italy

**Keywords:** COVID-19, SARS-CoV-2, Cinnamaldehyde, IL-1β, IL-6, Viral replication, Inflammation

## Abstract

**Background:**

The recent pandemic outbursts, due to SARS-CoV-2, have highlighted once more the central role of the inflammatory process in the propagation of viral infection. The main consequence of COVID-19 is the induction of a diffuse pro-inflammatory state, also defined as a cytokine storm, which affects different organs, but mostly the lungs. We aimed to prove the efficacy of cinnamaldehyde, the active compound of cinnamon, as an anti-inflammatory compound, able to reduce SARS-CoV-2 induced cytokine storm.

**Results:**

We enrolled 53 COVID-19 patients hospitalized for respiratory failure. The cohort was composed by 39 males and 13 females, aged 65.0 ± 9.8 years. We reported that COVID-19 patients have significantly higher IL-1β and IL-6 plasma levels compared to non-COVID-19 pneumonia patients. In addition, human mononuclear cells (PBMCs) isolated from SARS-CoV-2 infected patients are significantly more prone to release pro-inflammatory cytokines upon stimuli. We demonstrated, using in vitro cell models, that macrophages are responsible for mediating the pro-inflammatory cytokine storm while lung cells support SARS-CoV-2 replication upon viral infection. In this context, cinnamaldehyde administration significantly reduces SARS-CoV-2-related inflammation by inhibiting NLRP3 mediated IL-1β release in both PBMCs and THP-1 macrophages, as well as viral replication in CaLu-3 epithelial cells. Lastly, aerosol-administered cinnamaldehyde was able to significantly reduce IL-1β release in an in vivo lung-inflammatory model.

**Conclusion:**

The obtained results suggest the possible use of cinnamaldehyde as a co-adjuvant preventive treatment for COVID-19 disease together with vaccination, but also as a promising dietary supplement to reduce, more broadly, viral induced inflammation.

## Background

The COVID-19 pandemic, induced by SARS-CoV-2 infection, has highlighted once more the need for understanding the immune-inflammatory pathways involved in the regulation of the advancement of pulmonary infections. In fact, as for COVID-19, we lack specific pharmacological approaches targeting virus-induced inflammation, mainly because the underlying molecular mechanisms are still to be investigated. To date, based on recent reports by WHO a total of 13 variants of SARS-CoV-2 have been reported, divided between into variants of concern (VOCs) and variants of interest (VOIs) [1, 2]. However, despite the numerous adaptive mutations of the SARS-CoV-2, which resulted in the alterations of its pathogenic potential and challenges vaccine development, its pro-inflammatory attitude remains unchanged.

Different researchers have reported that COVID-19 patients show increased levels of pro-inflammatory cytokines, such as IL-6 and IL-1β [3, 4], indicating that their modulation might affect patients’ outcomes [5]. Despite the high number of reviews aiming to prove how the modulation of the NLRP3 (NOD-, LRR- and pyrin domain-containing protein 3) inflammasome, the main producer of IL-1β, might be beneficial in COVID-19 patients [5–7], only a few studies have been conducted to verify that hypothesis [8–10], and they provided limited data to support the use of NLRP3 inhibitor compounds in COVID-19 patients.

The NLRP3 is an intracellular sensor that detects a broad range of microbial motifs, leading to the formation and activation of the NLRP3 inflammasome, which is involved in the regulation of various diseases, from autoimmune disorders to cancer, representing a promising transversal target. During viral infection, NLRP3 inflammasome activation involves multiple cellular and molecular signaling resulting in the development of a consistent inflammatory state, which in turn promotes fibroblast proliferation, matrix deposition, and aberrant epithelial-mesenchymal function [11]. Accordingly, different NLRP3 inflammasome inhibitors have been reported to date, including the ones that directly inhibit the NLRP3 and those that act on its related signaling pathways [12]. Indeed, recently we identified a series of aryl sulfonamide derivatives (ASDs) and natural compounds with high potency and selectivity in inhibiting NLRP3 activation in vitro and in vivo [13, 14]. Among all the cited inhibitors, nutraceutical compounds might be of high impact for their low side effects in prolonged treatments. Notably, cinnamaldehyde (CINNA), the active compound of cinnamon (*Cinnamomum cassia* and *Cinnamomum verum*) which is often used as a flavoring condiment, is widely used in herbal medicine in China and Southeast Asia to treat inflammatory autoimmune diseases. Interestingly, it has been shown that CINNA acts on IL-1β release by modulating NLRP3 in both renal inflammation and rheumatoid arthritis in vivo [15, 16]. Moreover, it has been speculated that CINNA may also regulate oxidative stress [17], even if there are no effective studies to support this theory. Based on these concepts, CINNA treatment represents a promising therapeutic approach to modulate viral-induced lung inflammation. In this study, we aimed to assess CINNA efficacy by using SARS-CoV-2 infection as a viral activator of inflammation. To achieve our goal we: i) assessed the levels of pro-inflammatory cytokines as IL-1β and IL-6, in the plasma samples of severe COVID-19 patients compared to controls to prove the involvement of NLRP3 activation in the development of the disease; ii) evaluated the LPS-induced production of IL-1β and IL-6 from PBMCs of severe COVID-19 patients compared to healthy controls, to prove the effect of COVID-19 induced pro-inflammatory state, on the further activation of the NLRP3 axis; iii) the effects of CINNA in vitro in inhibiting IL-1β and IL-6 release as well as modulating viral replication; iv) the effects of CINNA in vivo in a lung-inflammatory model.

## Results

### Clinical overview of the enrolled subjects

In the present study, we enrolled 53 COVID-19 patients that were hospitalized for respiratory failure and required either invasive or non-invasive mechanical ventilation (NIMV), or only oxygen support. As resumed in Table 1, 75.5% of the patients were male and 24.5% female, with average age of 65.0 ± 9.8 years and average weight of 81.0 ± 15.5 kg. In line with the World Health Organization Ordinal Scale for Clinical Improvement [18], the level of severity was ranked from 5 to 9 based on the level of respiratory and vital support required at the time of assessment: 5 = oxygen supplementation; 6 = high-flow oxygen or NIMV; 7 = intubation and mechanical ventilation with pO2/FiO2 ≥ 150 or SpO2/FiO2 ≥ 200; 8 = mechanical ventilation pO2/FIO2 < 150 (SpO2/FiO2 < 200) or vasopressors; 9 = mechanical ventilation with pO2/FiO2 < 150 and vasopressors, dialysis, or extracorporeal membrane oxygenation (ECMO). Patients stratification according to the severity of infection is reported in Table 1.

**Table 1:** Description of the COVID-19 cohort

	**COVID-19 patients cohort** (*n* = 53, 100%)
**Gender**	
▪ Male	40 (75.5)
▪ Female	13 (24.5)
**Mean age (years ± SD)**	65.0 ± 9.8 (range 46—85)
**Mean weight (kg ± SD)**	81.0 ± 15.5 (range 47—130)
**Severity of infection**	
▪ 5	13 (24.5)
▪ 6	15 (28.3)
▪ 7	19 (35.8)
▪ 8	6 (11.3)
▪ 9	0 (0)

*SD* Standard deviation

### Increased blood plasma levels and increased PBMCs release of IL-1β and IL-6 were found in severe COVID-19 patients compared to controls

**Fig. 1:**
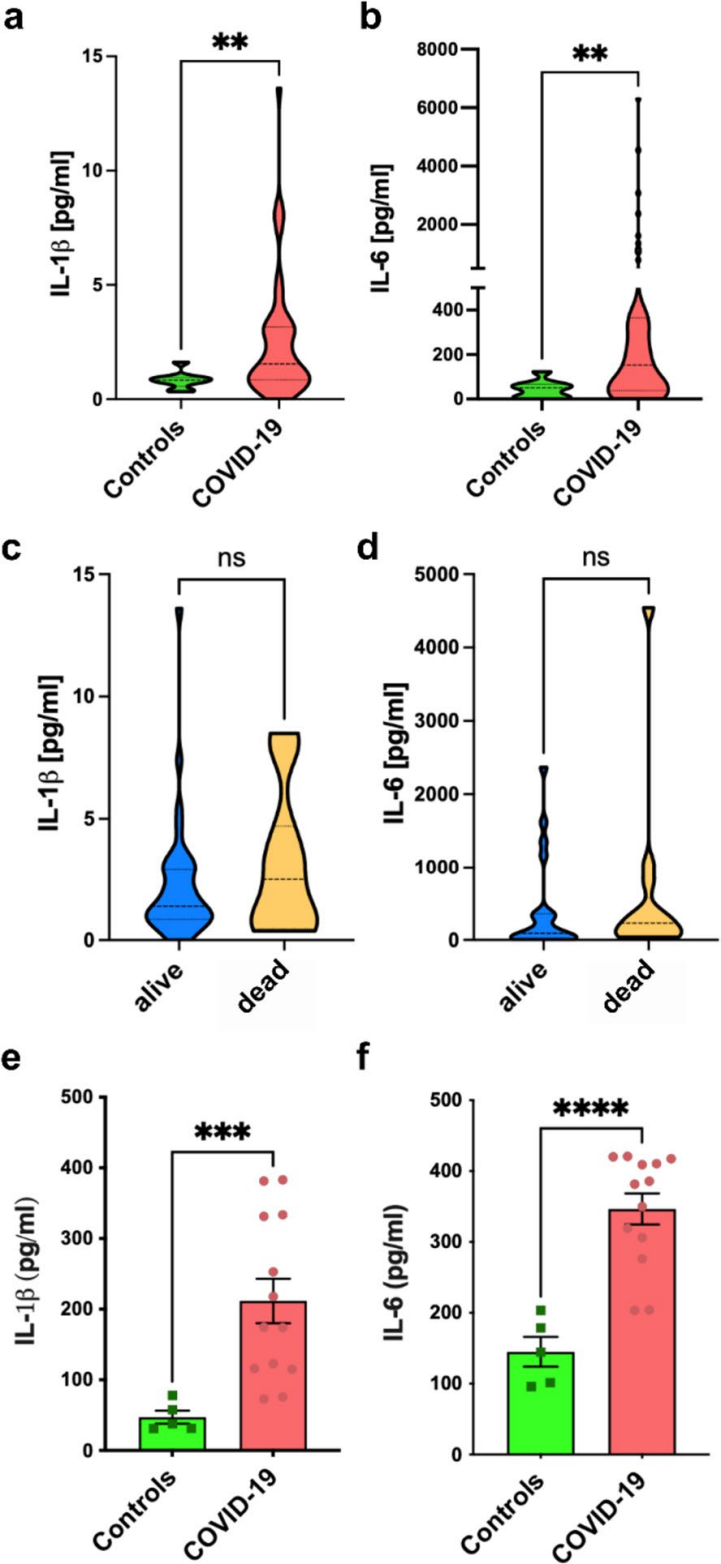
Increased IL-1β and IL-6 levels in COVID-19 patients’ plasma. **a** Violin plots showing IL-1β levels in subjects affected by non-COVID-19 pneumonia (controls, *n* = 9) vs COVID-19 patients (COVID-19, *n* = 53). **b** Violin plots showing IL-6 levels in controls (*n* = 9) vs COVID-19 patients (*n* = 37). **c** Violin plots showing IL-1β plasma levels in COVID-19 patients divided according to their outcome (alive *n* = 15; death *n* = 38). **D** Violin plots showing IL-6 plasma levels in COVID-19 patients divided according to their outcome (alive *n* = 11; death *n* = 17). **E** Bar graph showing IL-1β released after LPS + BzATP stimulation of PBMCs isolated from healthy subjects (controls, *n* = 5) and COVID-19 patients (COVID-19, *n* = 13). **F** Bar graph showing IL-6 released after LPS + BzATP stimulation of PBMCs isolated from healthy subjects (controls, *n* = 5) and COVID-19 patients (COVID-19, *n* = 13). ns = not significant, ** = *p* < 0,01, *** = *p* < 0,001, **** = *p* < 0,0001

In line with previous publications [19], we found significantly increased plasma levels of IL-1β and IL-6 compared to control hospitalized subjects with similar clinical severity but with non-COVID conditions (Fig. 1a, b). Numerically higher levels of IL-1β and IL-6 were found in patients who died compared to those who survived during the study period (Fig. 1c, d). At the same time, we evaluated if there were differences in the release of these cytokines also in vitro. Indeed, we isolated PBMCs from COVID-19 patients' and healthy controls and activated them with well-known inflammatory stimuli such as LPS + BzATP. Interestingly, following LPS and BzATP stimulation, we observed that PBMCs of COVID-19 patients significantly release more IL-1β and IL-6 compared to the control group (Fig. 1e, f). These results show that SARS-CoV-2 infection can trigger a specific systemic inflammation and suggest that the pro-inflammatory cytokines IL-1β and IL-6 play a central role in this pro-inflammatory systemic response.

### Macrophages mediate pro-inflammatory cytokine storm while lung cells support SARS-CoV-2 replication

**Fig. 2:**
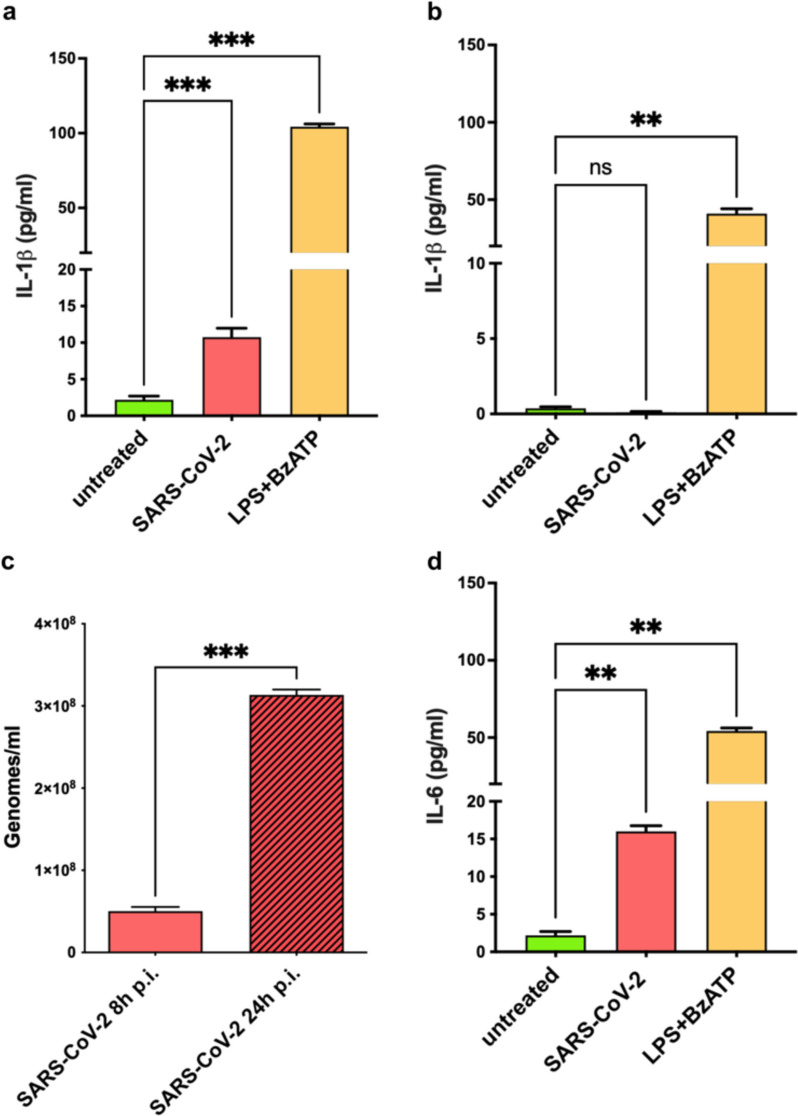
Differential effects of SARS-CoV-2 infection on THP-1 cells and CaLu-3. **a** Bar graph showing IL-1β release levels of THP-1 cells after 24 h from SARS-CoV-2 infection (*n* = 6) or LPS + BzATP stimulation (*n* = 3) compared to untreated cells (*n* = 4). **b** Bar graph showing IL-1β release levels of CaLu-3 cells after 24 h from SARS-CoV-2 infection (*n* = 3) or LPS + BzATP stimulation (*n* = 3) compared to untreated cells (*n* = 4). **c** Bar graph showing SARS-CoV-2 genomes in CaLu-3 cells at 8- and 24 h post-infection (h p.i.) (*n* = 2). **d** Bar graph showing IL-6 levels in THP-1 cells after 24 h from SARS-CoV-2 infection (*n* = 4) or LPS + BzATP stimulation (*n* = 2) compared to untreated cells (*n* = 3). ns = not significant, ** = *p* < 0,01, *** = *p < *0,001

To explore the relative contribution of immune and lung cells in the release of pro-inflammatory cytokines that follows SARS-CoV-2 infection, we evaluated the production of IL-1β in macrophages (THP-1) and lung (CaLu-3) cell lines. Both THP-1 and CaLu-3 cells were treated with a known inflammatory stimulus as LPS + BzATP or infected with the SARS-CoV-2 virus. Then, the cellular supernatants were tested for cytokines release and viral replication. We highlight that only THP-1 cells showed a significant increase in IL-1β production after viral infection, while CaLu-3 cells were completely unaffected (Fig. 2a, b). Remarkably, we found that IL-1β production occurred in both cell lines when stimulated with an NLRP3 activator (LPS + BzATP), thus indicating a selective inability of the SARS-CoV-2 virus to induce the same effect in CaLu-3 cells (Fig. 2a, b). Nevertheless, the main consequence of viral infection on CaLu-3 cells is the replication of the virus, instead of the promotion of a pro-inflammatory response, as shown by the progressive viral genome increase detected in SARS-CoV-2 infected CaLu-3 (Fig. 2c). Accordingly, THP-1 cells also showed an increased release of IL-6 upon SARS-CoV-2 infection (Fig. 2d), further supporting their potential role in modulating the pro-inflammatory cytokine storm observed in COVID-19 patients. These data indicate that while lung cells are important for virus infection and replication, activation of immune-inflammatory cells is pivotal in triggering the pro-inflammatory cascade that follows the infection.

### Cinnamaldehyde treatment strongly reduces IL-1β and IL-6 secretion together with SARS-CoV-2 replication

**Fig. 3:**
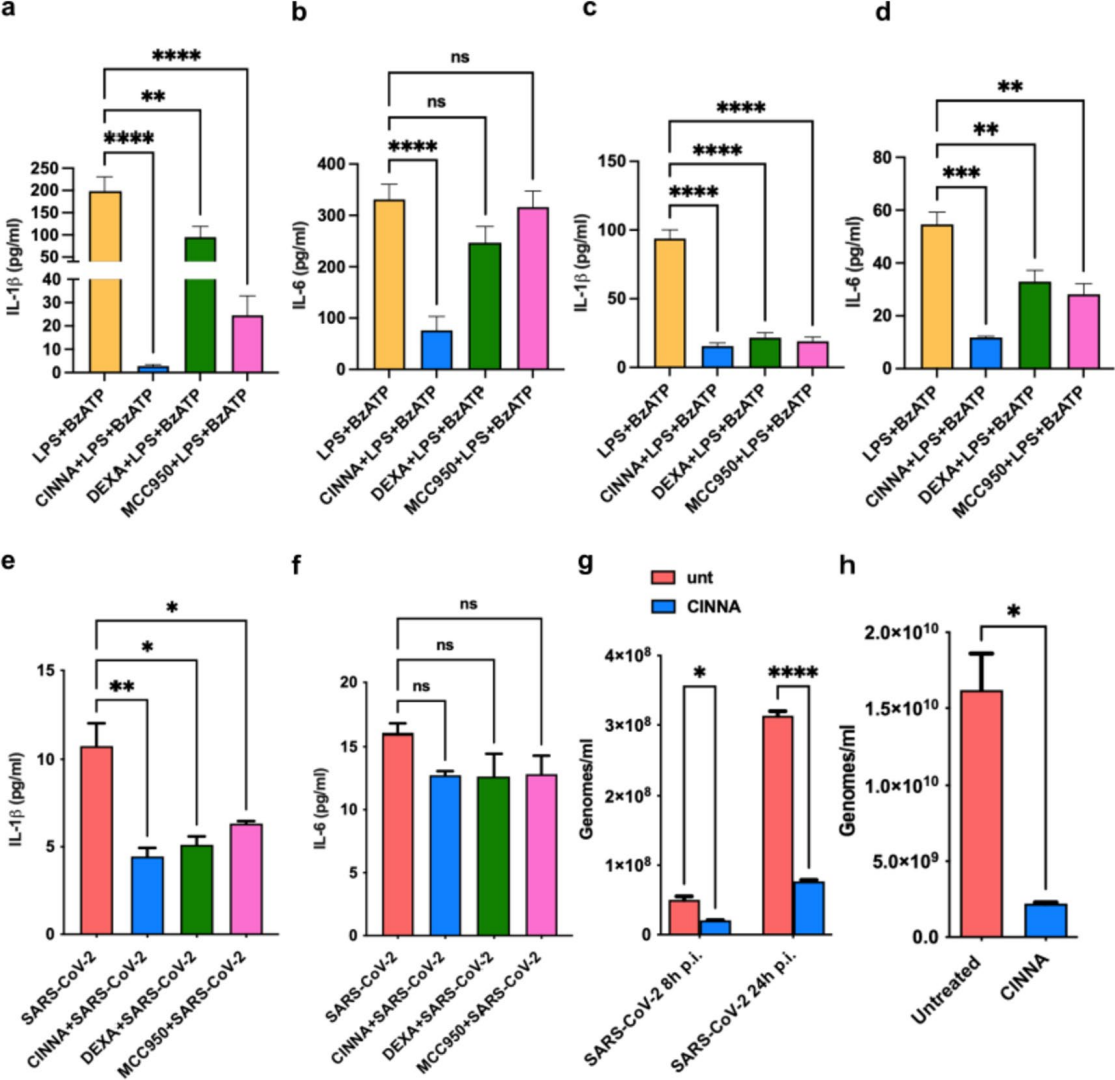
Effects of cinnamaldehyde on SARS-CoV-2 induced inflammation and viral replication. **a** Bar graph showing IL-1β levels released by PBMCs of COVID-19 patients (*n* = 13) after LPS-BzATP stimulation and the pretreatment with different compounds: cinnamaldehyde (CINNA), dexamethasone (DEXA) and MCC950. **b** Bar graph showing IL-6 levels released by PBMCs of COVID-19 patients (*n* = 13) after LPS-BzATP stimulation and the pretreatment with different compounds: CINNA, DEXA, and MCC950. **c** Bar graph showing IL-1β levels released by THP-1 after LPS-BzATP stimulation and the pretreatment with different compounds: cinnamaldehyde (CINNA), dexamethasone (DEXA) and MCC950 (*n* = 3). **d** Bar graph showing IL-6 levels released by THP-1 after LPS-BzATP stimulation and the pretreatment with different compounds: CINNA, DEXA, and MCC950 (*n* = 3). **e** Bar graph showing IL-1β levels released by THP-1 after SARS-CoV-2 infection (*n* = 6) and the pretreatment with different compounds (*n* = 4): CINNA, DEXA, and MCC950. **f** Bar graph showing IL-6 levels released by THP-1 after SARS-CoV-2 infection (*n* = 2) and the pretreatment with different compounds: CINNA (*n =* 4), DEXA (*n* = 2), and MCC950 (*n* = 2). **g** Bar graph showing SARS-CoV-2 genomes in CaLu-3 cells 8- and 24-h post-infection (h p.i.) with or without the pretreatment with CINNA (*n* = 2). **h** Histograms showing SARS-CoV-2 replication rate in VeroE6 cells 48 h after infection with or without the pretreatment with CINNA (*n* = 2). ns = not significant, * = p < 0,05, ** = *p* < 0,01, **** = *p < *0,0001

Besides the effects of vaccines and monoclonal therapies, there is still a lack of drugs that allow an efficient pharmacological approach to COVID-19 patients. Therefore, we aimed to identify a compound that might counteract the previously observed effects of SARS-CoV-2 infection on pro-inflammatory cytokines release. We identified the natural compound cinnamaldehyde (CINNA) as a promising candidate since it has been shown to act on NLRP-3 inflammasome reducing IL-1β release [15, 16, 20]. At the same time, we tested the effects of dexamethasone on these pathways, which nowadays represents one of the standard treatments for severe COVID-19. Also, the known NLRP3 inhibitor MCC950 was used as a control. Thus, we evaluated the release of proinflammatory cytokines in vitro, in the supernatant of PBMCs isolated from COVID-19 patients and activated with LPS + BzATP. Notably, CINNA pretreatment strongly decreased LPS + BzATP-induced inflammation in COVID-19 patients’ PBMCs, by inhibiting the SARS-CoV-2-enhanced proinflammatory state (Fig. 3a, b). The effects of CINNA were even more pronounced when compared with dexamethasone and MCC950, both of which were ineffective in diminishing IL-6 release (Fig. 3b). We subsequently tested the direct effect of CINNA pretreatment on LPS-induced inflammation and SARS-CoV-2 infection on THP-1 cells. THP-1 were then activated with LPS + BzATP or infected with SARS-CoV-2 and treated with our molecules of interest. Intriguingly, in this context, CINNA strongly reduced IL-1β and IL-6 secretion induced by LPS comparable to dexamethasone and MCC950 (Fig. 3c, d), as well known from the literature [21, 22]. However, after viral infection, CINNA could reduce IL-1β levels but not significantly counteract the IL-6 one (Fig. 3e, f). Similarly, dexamethasone and MCC950 only showed their efficacy in counteracting NLRP3 activation (Fig. 3e, f). Lastly, we investigated the effects of CINNA in affecting SARS-CoV-2 viral replication by using two distinct in vitro cell models, the aforementioned CaLu-3 and the VeroE6, which are considered the gold standard cell line to study viral replication for SARS-CoV-2. As shown in Fig. 3g, CINNA pretreatment lessens SARS-CoV-2 viral replication threefold at both 8- and 24-h post-infection in CaLu-3 cells. A similar effect was also observed on VeroE6 cells 48 h after infection (Fig. 3h).

### Aerosol administration of CINNA reduces IL-1β secretion in an in vivo lung-inflammatory model

**Fig. 4:**
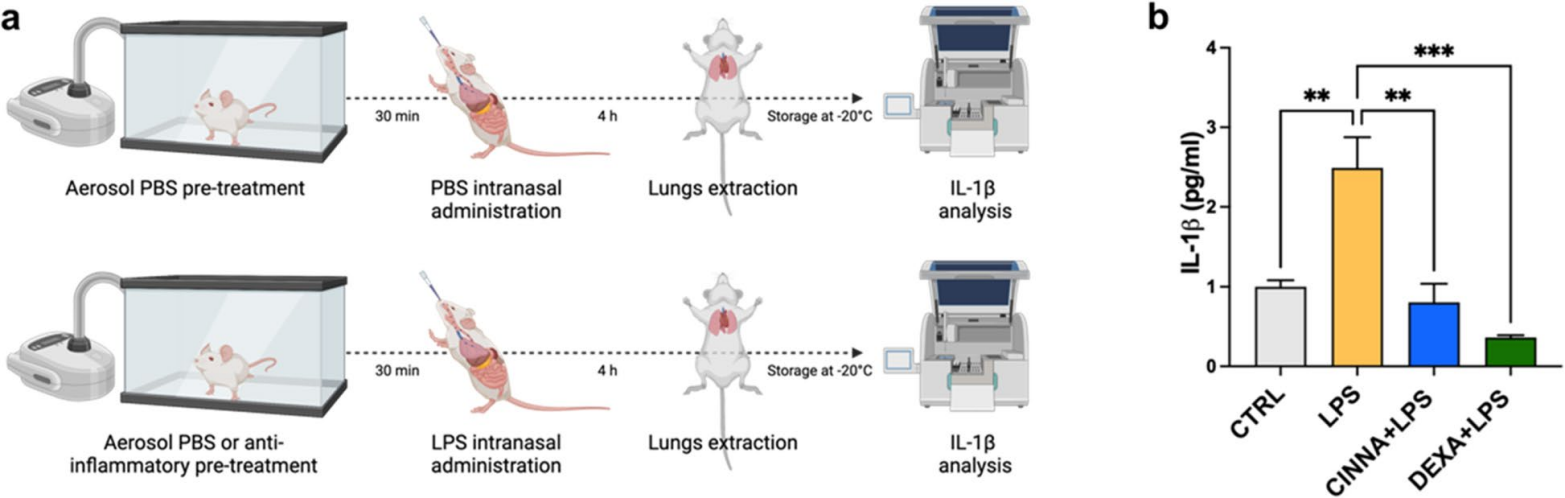
Effects of Cinna-Sol® on IL-1β release in an in vivo lung-inflammatory model. **a** Schematic representation of the experimental design. The C57BL/6 J-WT mice were divided into two groups: a control group receiving PBS treatment (CTRL), and a group subjected to intranasal LPS-induced lung inflammation. Within the LPS group, there were three sub-groups: PBS inhalation (LPS), CINNA inhalation (CINNA + LPS), and DEXA inhalation (DEXA + LPS) received as aerosol pre-treatments. Following lung extraction, the release of IL-1β from samples was analyzed by ELISA assay. **b** Bar graph showing ELISA results of IL-1β levels released by lungs of C57BL/6 J mice in control condition (CTRL) (*n* = 15), after LPS treatment (LPS) (*n* = 17) or pre-treated with aerosol inhalation of CINNA (CINNA + LPS) (*n* = 5) or DEXA (DEXA + LPS) (*n* = 4). ** = *p* < 0,01, *** = *p* < 0,001

Next, we aimed to assess the effects of CINNA pretreatment on the release of IL-1β in an in vivo model of LPS-induced lung inflammation. To induce direct inflammation in lungs, we performed intranasal administration of LPS into C57BL/6 J mice. Mice were categorized into two sets: the control group that received intranasal administration of PBS (CTRL), and one that underwent intranasal induction of lung inflammation using LPS. Within the LPS-exposed group, we tested the anti-inflammatory capacity of CINNA, giving it by aerosol as pretreatment before LPS intranasal injection (CINNA + LPS). As a control, we performed aerosol administration of DEXA (DEXA + LPS) (Fig. 4a). As expected, LPS treatment (LPS) was able to increase IL-1β release compared to the control group (CTRL), while both treatments with CINNA and DEXA exhibited a large inhibitory effect on the levels of IL-1β release induced by intranasal LPS administration (Fig. 4b). These findings suggest that CINNA has a pronounced ability to mitigate the release of IL-1β in the context of LPS-induced lung inflammation not only in vitro but also in vivo.

## Discussion

Chronic inflammation is one of the main hallmarks of several pulmonary diseases and pathogens infectious. Over the years, research has made significant strides in the treatment of respiratory diseases, due to bacterial and viral infections. However, a treatment for the reduction of pro-inflammatory cytokine storm induced by pathogens, with minimal or no side effects, is still an important goal to be reached. Here, we developed a model of the pathogen-induced pro-inflammatory condition by using SARS-CoV-2 infection, and we evaluated the anti-inflammatory properties of the natural compound CINNA.

First, we confirm previously reported data showing that COVID-19 patients show increased plasma levels of IL-1β and IL-6 compared to pneumonia patients non-SARS-CoV-2 related [23, 24]. Interestingly, our data highlight that PBMCs of COVID-19 patients are more prone to release proinflammatory cytokines, such as IL-1β and IL-6, upon stimulation, thus indicating that virus infection might prime and boost the systemic inflammatory responses exposing patients to inflammation-related clinical impairment. Furthermore, we have shown that SARS-CoV-2 infection elicits IL-1β and IL-6 release only in macrophages, while lung cells are the site for viral replication. These results suggest that the preventive use of anti-inflammatory compounds might result in a beneficial effect on disease resolution, by reducing the diffuse COVID-19 pathology.

Several studies have shown the promising anti-inflammatory effects of some phyto-compounds, used to reduce inflammation in different conditions [25]. Phyto-compounds, extracted from plants, are generally characterized by reduced side effects in long-term treatments and low production costs. In addition, complementary approaches based on natural compounds are gaining increasing attention due to superior patient compliance compared to traditional synthetic drugs [26–28]. Our data have shown that CINNA administration can reduce LPS-induced IL-1β and IL-6 release in human PBMCs, and consistently also in THP-1 macrophages. However, CINNA treatment, as well as dexamethasone and MCC950, were able to reduce only SARS-CoV-2-induced IL-1β release in THP-1 macrophages, leaving SARS-CoV-2-induced IL-6 secretion unaffected. However, CINNA treatment, as well as dexamethasone and MCC950, were able to significantly reduce the release of IL-1β induced by SARS-CoV-2 in THP-1 macrophages, while having only a moderate effect on SARS-CoV-2-induced IL-6 secretion.

It is well-known that IL-1β possesses a broad spectrum of pro-inflammatory properties, including the ability to induce the synthesis of other pro-inflammatory cytokines and chemokines (such as TNF-*α*, IL-6, and IL-8) [29, 30]. Nevertheless, in the cited experiment performed on THP-1 macrophages, CINNA, dexamethasone, or MCC950 pre-treatment only moderately affect IL-6 release, suggesting that in SARS-CoV-2 infection, IL-6 release might be independent of IL-1β activation*.* Therefore, considering that pre-treatment with the employed compounds only had a modest impact on IL-6 release in THP-1 macrophages infected with SARS-CoV-2, it is plausible to suggest that in this condition, the release of IL-6 may occur independently of IL-1β activation.

Several studies have reported that the use of anakinra, a dual blocker of IL-1α and IL-1β, in COVID-19 patients reduced the mortality risk, especially in the presence of signs of hyperinflammation [8, 31–33]. However, facing the severity of COVID-19, it is imperative to identify treatments that are easily accessible to the population who do not need to be hospitalized [34]. In this scenario, CINNA, as an easy-to-assume nutraceutical, represents a promising preventive approach to be used to prevent the appearance or aggravation of COVID-19 respiratory symptoms. Indeed, our in vivo lung inflammatory model demonstrates a significant reduction in IL-1β release following pre-treatment with CINNA. In addition, our data proved that CINNA pre-treatment reduces the viral replication rate in lung cells, suggesting that CINNA might provide beneficial effects by down-regulating the synthesis of IL-1β also by reducing viral spreading. The possible wide-spectrum application of CINNA increases its value if evaluated also within the long COVID. Indeed, different studies that have analyzed the numerous symptoms of long COVID, have evidenced that inflammation, in different districts of the organism, plays a key role in the persistence of the pathology [35–37]. Therefore, it is crucial to find a therapy that can reduce chronic inflammation and, consequently, the long-term effects of SARS-CoV-2 infection.

Inflammation is not an exclusive characteristic of COVID-19 but is also present in many other forms of pneumonia caused by various pathogens. Therefore, its application could also be relevant in the treatment of different types of pneumonia. Hence, CINNA represents a promising dietary supplement to reduce the pro-inflammatory effects due to pathogens-induced pulmonary infections.

## Conclusions

Chronic inflammation plays a significant role in respiratory diseases and pathogen-induced infections. This study explored the anti-inflammatory properties of CINNA, a natural compound, in the context of SARS-CoV-2 infection. Analysis of COVID-19 patients revealed heightened serum levels of IL-1β and IL-6, suggesting a pivotal role of these cytokines in the systemic inflammatory response associated with the disease. Our in vitro findings highlighted the potential of CINNA as a dual-action agent, able to both reduce the release of pro-inflammatory cytokines, particularly IL-1β, and attenuate viral replication in lung cells. This proposes CINNA as an appealing candidate for managing respiratory symptoms and reducing viral spread. In an in vivo lung inflammation model, we reported that aerosol-administered CINNA effectively reduced IL-1β release, further sustaining its potential for translation into the treatment of inflammatory lungs diseases in humans. The results support the use of CINNA as a promising co-adjuvant treatment strategy alongside vaccination to mitigate the pro-inflammatory effects associated with pulmonary infections, including COVID-19, as well as a potential dietary supplement to address extensive viral-induced inflammation. Although additional investigation is needed to explore the broader application of CINNA in human respiratory diseases caused by various pathogens, these results underscore the importance of this study as a promising starting point.

## Methods

All the reagents used have been purchased from Merck Millipore (Burlington, USA) if not otherwise specified.

a *Subjects’ enrollment*

The present analysis included blood samples of patients from the “Pro-thrombotic status in patients with SARS-CoV-2 infection” (ATTAC-Co) study that was approved by the local Ethic Committee CE-AVEC (Comitato Etico di Area Vasta Emilia Centro, Bologna, Italy) [38]. The ATTAC-Co study is an investigator-initiated, prospective, single-center study recruiting consecutive patients admitted to the hospital (University Hospital of Ferrara, Italy) because of respiratory failure due to COVID-19 between April and May 2020. Inclusion criteria are detailed elsewhere [39]. Briefly, patients were (i) age > 18 yr; (ii) confirmed SARS-CoV-2 infection; (iii) hospitalized for respiratory failure; (iv) need for invasive or non-invasive mechanical ventilation or only oxygen support. SARS-CoV-2 infection was confirmed by RT-PCR assay (Liaison MDX; Diasorin) from a nasopharyngeal swab specimen. Respiratory failure was defined as a PaO2/FiO2 (P/F) ratio ≤ 200 mmHg. Clinical management was in accordance with current guidelines and specific recommendations for the COVID-19 pandemic by Health Authorities and Scientific Societies. A control group of patients, hospitalized in the same period and hospital settings with acute respiratory failure due to respiratory/cardiovascular acute conditions not related to SARS-CoV-2, was also included. This analysis was performed on blood samples collected just after admission. Ethics approval has been obtained. All patients gave their written informed consent. In case of unconsciousness, the informed consent was signed by the next of kin or legally authorized representative. The study is registered at www. clinicaltrials.gov with the identifier NCT04343053.

b *Plasma collection and PBMCs isolation*

Plasma was collected by centrifuging patients’ blood at 1000 × g for 30 min, the obtained aliquots were stored at -80 °C.

Peripheral blood mononuclear cells (PBMCs) were isolated from blood samples of a subgroup of COVID-19 patients and healthy donors. Ficoll-Paque density gradient centrifugation was used for PBMC separation. Blood was diluted with an equal volume of PBS. Diluted blood was layered over 4 mL of the Ficoll-Paque PLUS. Gradients were centrifuged at 400 × g for 30 min at room temperature in a swinging-bucket rotor without the brake applied. The PBMCs interface was carefully removed by pipetting and washed with PBS by centrifugation at 250 × g for 10 min then a total of 5 × 10^5^ cells/well were seeded in 24-well tissue culture plates in RPMI medium supplemented with 10% FBS, 100 U/mL penicillin, and 100 mg/mL streptomycin. PBMCs were maintained in a 5% CO_2_ incubator at 37 °C.

c. *Cell cultures*

The human leukemia monocytic cell line THP-1 was grown in RPMI medium supplemented with 10% FBS, 100 U/mL penicillin, and 100 mg/mL streptomycin. THP-1 cells were stimulated by 100 ng/mL PMA overnight to differentiate into macrophages. The human lung cancer (CaLu-3) and human airway epithelial (VeroE6) cell lines were grown in Eagle’s minimal essential medium (MEM) with nonessential amino acids supplemented with 10% FBS, 100 U/mL penicillin, 100 mg/mL streptomycin and 2 mM L-glutamine. All cells were grown in a 5% CO_2_ incubator at 37 °C. THP-1 (TIB-202), CaLu-3 (HTB-55) and VeroE6 (CRL1586) cells were purchased from ATCC.

d. *Inflammasome activation assays *in vitro

Human PBMCs and THP-1 cells were seeded at 5 × 10^5^ cells/well and 3 × 10^5^ cells/well respectively in 24 well plates. The following day cells were stimulated with LPS (1 μg/mL) for 3 h and then with 2′(3′)-O-(4-Benzoylbenzoyl) adenosine 5′-triphosphate (100 μM) for 30 min.

e * SARS-CoV-2 Propagation and Infection*

SARS-CoV-2 was isolated from a nasopharyngeal swab retrieved from a patient with COVID-19 (Caucasian man of Italian origin, genome sequences available at GenBank (SARS-CoV-2-UNIBS-AP66: ERR4145453). This SARS-CoV-2 isolate clustered in the B1 clade, which includes most of the Italian sequences, together with sequences derived from other European countries and the United States. As previously described, the viral titer was determined by plaque assay in Vero E6 cells [40]. SARS-CoV-2 manipulation was performed in the BSL-3 laboratory of the University of Ferrara, following the biosafety requirements and accordingly with the Institutional Biosafety Committee. THP-1, CaLu3 and VeroE6 cell lines were exposed to SARS-COV-2 as previously described [41] in the presence or absence of modulatory interventions as detailed below. Cells supernatant was collected at 8-, 24-, or 48 h post-infection and after the different pharmacological treatments.

f *Viral RNA Detection*

RNA extraction was performed 8 and 24 h post-infection (hpi) with MagMAX viral/pathogen nucleic acid isolation kit (Thermo Fisher, Milan, Italy), a kit for the recovery of RNA and DNA from the virus. SARS-CoV-2 titration was obtained by TaqMan 2019nCoV assay kit v1 real-time qPCR (Thermo Fisher, Milan, Italy).

g *In vivo lung-inflammatory model*

12-weeks old C57BL/6 J mice were injected intranasally with LPS: 10 µg of LPS diluted in 25µl of sterile PBS for each mouse. After 4 h the mice were sacrificed by cervical dislocation and their lungs were collected and stored at -20 °C until further processing. To control mice were administered 25µl of sterile PBS by intranasal injection.

h * ELISA*

Plasma, PBMCs, THP-1, and CaLu-3 cell cultures supernatants were assayed for human IL-1β or IL-6 using either ELISA kits or *Ella*™ arrays following the manufacturer’s instructions (R&D Systems, Minneapolis, USA). Mice lungs were homogenized using TissueRuptor™ (Qiagen, Hilden, Germany) with an optimized lysis buffer (300 mM sucrose, 1 mM K2HPO4, 1 mM MgSO4, 5.5 mM D-glucose, 20 mM HEPES, 0.5% IGEPAL and supplemented with protease inhibitor cocktail) following the manufacturer’s instructions. Following a centrifugation of 12,000 × g at 4 °C for 15 min, the supernatant was collected and filtered with a 0,45 µm filter. The obtained specimens were assayed for mouse IL-1β using *Ella*™ arrays following the manufacturer’s instructions (R&D Systems, Minneapolis, USA).

i. *Pharmacological treatments*

Cells were pre-treated before viral infection with the following compounds: MCC950 1 mM for 30 min, cinnamaldehyde (CINNA) 50 µM for 30 min, and dexamethasone (DEXA) 100 nM for 1 h. For the in vivo model, mice were pre-treated 30 min before LPS administration with the following compounds by aerosol administration: Cinna-Sol® (CinnaPharm, Italia) 1 vial each mouse, and dexamethasone (DEXA) 25 mg/kg diluted in 5 ml of PBS.

j *Statistical analysis*

The data were analyzed by Prism 6 (GraphPad). Normal distributions were assessed using the Shapiro–Wilk test. Student’s t-test for unpaired data with Welch’s correction was used to compare two datasets with normal distribution, while the Mann–Whitney test was used for non-normal datasets. Multiple comparisons of ≥ 3 experimental groups were performed using Brown-Forsythe and Welch ANOVA one-way test by comparing all the groups with the untreated or with the control ones (Dunnett’s T3 multiple comparison test). *P* < 0.05 indicates statistical significance.

## Data Availability

The datasets used and/or analyzed during the current study are available from the corresponding author upon reasonable request.

## Abbreviations

Bz-ATP: 2ʹ(3ʹ)-O-(4-Benzoylbenzoyl)adenosine-5ʹ-triphosphate tri(triethylammonium) salt
CINNA: Cinnamaldheyde
DEXA: Dexamethasone
IL-1β: Interleukin 1β
IL-6: Interleukin 6
IL-8: Interleukin 1β
LPS: Lipopolysaccharides
NIMV: Non-invasive mechanical ventilation
NLRP3: NOD-like receptor 3
PBMCs: Peripheral blood mononuclear cell
TNF-α: Tumor Necrosis Factor α
